# Editorial: Public Mental Health Policy, Mental Health Promotion, and Interventions Which Focus on the Social Determinants of Mental Health

**DOI:** 10.3389/fpubh.2016.00285

**Published:** 2016-12-26

**Authors:** Samantha Battams

**Affiliations:** ^1^Torrens University Australia, Adelaide, SA, Australia; ^2^Southgate Institute for Health, Society and Equity, Flinders University, Adelaide, SA, Australia; ^3^Health Outcomes International, Glynde, SA, Australia

**Keywords:** mental health, public mental health, mental health intervention, mental health promotion, psychiatric disability, human rights

**Editorial on the Research Topic**

**Public Mental Health Policy, Mental Health Promotion, and Interventions Which Focus on the Social Determinants of Mental Health**

The 2006 United Nations convention on the rights of persons with disabilities (CRPD) (1) enshrined a range of rights, with the aim of ensuring independence and full social inclusion for people with disabilities. The convention is based on a “social model” of disability, which underscores the role of social environments and systems in contributing to disability; it thus moves beyond a “medical model” where the focus is on individual deficits associated with disability, or a “charity model” that highlights need and dependency of people with disabilities (2, 3). The CRPD covers people with disability arising from mental disorders, who are particularly vulnerable to rights violations (4).

The human rights outlined in the CRPD cover “positive rights” such as access to employment, social, and health services, as well as so-called “negative rights” such as the right to refuse treatment and the deprivation of liberty (5). However, it has been noted that there is an undue attention on “negative rights” in treatment services (5), and limited capacity for the law to guarantee “positive rights” (2). Battams and Henderson note (5) that:

it has been argued that there is a much greater focus on ‘negative rights’, or the civil/political rights to refuse treatment and freedom from detention, rather than ‘positive’ rights such as the social/economic right to health care and access to treatment (6–8). Similarly, there is a greater focus on ‘procedural rights’ (i.e., due process) in relation to people with psychiatric disability, rather than ‘substantive rights’ such as the right to health (9–11).

They also found that in their own study on the implementation of the CRPD in Australia, there was a focus on rights in relation to treatment services, and a lack of focus on citizenship rights in relation to social inclusion and access to housing and other social determinants (2).

In this context, this research topic sought to attract articles on public mental health policies, mental health promotion, and interventions with a particular focus on the social determinants of mental health and the right to health for people with mental health conditions (i.e., positive rights). In particular, the criteria for articles for this special research topic were those which considered the following:

(1) Policy and interventions that promote the “right to health” and access to mental health and physical health care for people with mental health conditions.
(2) Policy and interventions that address access to health issues for people with comorbidity between mental health and other health conditions.
(3) Policy and interventions that are oriented toward mental health promotion and/or address the social determinants of health (e.g., housing, social support, employment, culture, stigma/discrimination, and access to services).

There are four articles in this research topic, two of which explored issues linked to access to mental health services. Forouzan et al. considered mental health service responsiveness in Iran. Sweeney et al. explored access to mental health services according to socioeconomic status in South Australia. In addition, the article by Flammer and Steinert was based on research on negative rights for people with mental disorders in treatment services. Finally, Xiang et al. considered the mental health of recently bereaved and non-bereaved earthquake survivors.

Forouzan et al. adapted a framework for measuring health service responsiveness developed by the World Health Organization (WHO) (12), and applied this to mental health outpatient services in Tehran. Health service responsiveness refers to non-medical aspects of the health system, including processes for care, social and physical care environments, and access to support during care. Specific aspects of the WHO framework for measuring service responsiveness include respect for dignity; respect for autonomy; respect for confidentiality; prompt attention; quality of basic amenities; access to social support networks during care; and choice of care provider.

Lack of prompt attention to mental health patients (particularly in emergency departments) has become a concern in under-resourced mental health systems, especially where housing and associated psychosocial support may be limited. For example, patients have waited in an emergency department for up to 4–5 days in less than optimum physical environments before receiving treatment.^1^^,^^2^ Long hospital waiting periods have been associated with other concerns such as respect for dignity and involuntary medication. The application of the WHO health service framework to the mental health service environment fits in with a human rights-based approach to health and addressing “negative rights,” such as the deprivation of liberty. A human rights-based approach to health services (13) includes the “PANEL” principles of

- Participation in one’s own development
- Accountability of duty bearers to rights-holders
- Non-discrimination and prioritization of vulnerable groups
- Empowerment of rights-holders
- Legality; the express application of a human rights framework.

The paper by Sweeney et al. demonstrates the link between lower socioeconomic status and increased use of hospitals for mental health problems, particularly Emergency Departments, in a metropolitan region of South Australia; these lower socioeconomic status patients had less contact with GPs, family, and other social support in the community. This paper highlights the challenges for the implementation of the CRPD for people with psychiatric disability and the limits of the law in terms of guaranteeing positive rights. It also suggests that equity considerations should be taken into account when introducing mental health interventions, including those aligned to a social model of disability. Under a human rights-based approach to service delivery, population groups not having ready access to community-based mental health services and social support would ideally be prioritized as vulnerable groups.

The article by Xiang et al. reveals the need for prompt mental health resources and social support networks in the event of natural disasters such as earthquakes, especially for bereaved survivors who may have lost loved ones who would usually form part of support networks to assist coping with such events. It also suggests that emotional states, associated with disasters and personal tragedy, may influence findings on measures of personality traits and coping styles, challenging trait approaches to mental health problems. This article points to the need for mental health services to form a critical part of humanitarian and disaster responses – along with sustainable development.

Flammer and Steinert explore “negative rights” in treatment services in Germany, following the CRPD. They researched involuntary admissions in treatment services, and their association with “last resort” involuntary medication and other coercive measures such as physical restraint. They found that involuntary medication was rarely used, but that seclusion and mechanical restraint were more common coercive measures. Changes in the use of involuntary medication were associated with changes to legislation (following the CRPD). This is an important article as it provides previously unconsidered and extensive data on “negative rights” following the introduction of the CRPD and changes to German law. However, numbers reported for involuntary medication and other coercive measures appear to be very small, which may also possibly suggest under-reporting. A previous Norwegian study has shown that restraint and involuntary medication were used equally on an acute psychiatric ward (14). Another Dutch study showed that there were no differences in involuntary medication after a national program to reduce seclusion in psychiatric hospitals; this study noted a blurring of distinction between persuasion and coercion which may lead to under-reporting on involuntary medication usage (15).

In a further contribution on this topic, Steinert et al. (16) have published a systematic review that found only 11 research studies that referred to the CRPD for people with mental disorders (leading to psychiatric disability), including a small study previously undertaken by myself and a colleague (5) on the implementation of the CRPD in Australia.

Australia has recently introduced the National Disability Insurance Scheme (NDIS) which is a step toward realizing a broader range of rights for people with psychiatric disability, such as those outlined in the CRPD with disability. This scheme will be crucial for the realization of the CRPD and particularly social inclusion. The scheme is about promoting social inclusion and access to positive rights for people with psychiatric disability and other people with disabilities. There are few schemes like it around the world, and I mention it here as it is relevant to the criteria established for this research topic. Early feedback on the scheme suggests avenues for further research. In addition, it calls itself a “world leader” as it aims for “balancing individual responsibility and family care with government support and the disruptive innovation of the marketplace” (17). Funding for the scheme comes from a national tax, part of the Medicare levy, used for Australia’s universal health care scheme. The 3-year trial phase of the NDIS was just completed (September 2016), and half of its participants received disability support services *for the first time* (18).

This NDIS scheme “supports people with a permanent and significant disability that affects their ability to take part in everyday activities”.^3^ Supports aim to assist people with disabilities to achieve independence and goals related to community participation, education, employment, and health and wellbeing. Under the NDIS, an overarching concern is “access to services” for people with psychiatric disability. The NDIS is also being introduced in the context of changes in the primary care sector and a push for personally controlled e-health records.

The NDIS is focused on “consumer directed care” or substitute decision making – carers, the NDIS, or registered plan managers can make choices about NDIS plans (which involve goals and supports). Substitute decision making is particularly relevant to people with psychiatric disability during periods of episodic illness. The CRPD supports substitute decision making and supportive decision making, which Australia recognises,^4^ however in reality there are few examples of its implementation for people with psychiatric disability. It also may prove difficult as the nature of psychiatric episodes may mean that people are isolated or estranged and have fewer family/friends around willing to act as substitute or supportive decision makers.

The aim of the NDIS was to ensure consumer choice and control for support services for those with a permanent disability, as well as early intervention. Service users (with the help of supportive or substitute decision makers) choose (1) service providers, (2) how services are provided, and (3) where they are provided. The NDIS involves service agreements between service providers and service users or substitutes decision makers (e.g., family carers), as well as the NDIS plan. The service agreement includes “responsibilities” of the service users and “expectations” of service providers.^5^ There are three tiers of funding under the NDIS, with tier 3 being about individualized support packages of support; this is the focus for people with a psychiatric disability.

While generally highly supportive of the NDIS, a number of mental health organizations and individual advocates have flagged some potential challenges for the NDIS providing support to people with a psychiatric disability as intended. These will have lessons for the full implementation of the NDIS and other similar schemes around the world, which aim to provide a comprehensive approach for people with a range of disabilities. Concerns raised by the Mental Illness Fellowship of Australia (19), the Mental Health Council of Australia (20), and its former CEO John Mendoza (21) include the following:

- *Disability versus recovery*: the NDIS is largely geared toward people with permanent disability. This potentially conflicts with the focus on “early intervention” and “recovery” from mental illness in Australian National Mental Health Policy. There is thus concern that the scheme overlooks a recovery model, may create perverse incentives and encourage dependence. Conversely, some services flagged for inclusion under the NDIS come under the scope of “early intervention” and temporary support rather than ongoing disability support, which may be required for those most affected by their illness.
- *Episodic nature of illness*: initially, access criteria for NDIS services did not take into account the episodic nature of mental illness. Episodic mental illness may impact upon capacity to exercise choice and control under the NDIS. People may require substitute decision makers during such times (but not at other times). There is potential for confusion between episodic mental illness and underlying disability.
- *Lack of substitute decision makers*: due to the nature of some mental health conditions, people with psychiatric disability may lack carers or family members to be substitute decision makers.
- *Confusion about numbers and concerns about access for people with psychiatric disability*: the Mental Health Council of Australia argued that the Productivity Commission estimates that there are 60,000 people who would require tier 3 support and 10% who would require intensive support “lacks any credibility and vastly underestimates the level of need in the community” (20). Similarly, the Mental Illness Fellowship of Australia claims that “half of the people with severe mental illness are not engaged with the system” (19).
- *Lack of access to NDIS services*: for people who do not “opt in” or are not deemed to have sufficient disability. Those most disabled by their mental health condition may lack insight into their illness. People with mental health conditions may not have a lifelong disability or may not accept that they have one. The notion of permanent impairment is also unclear due to episodic illness which may entail fluctuating needs.
- Systems issues: these include pricing of services, delayed payments to providers, workforce issues and lack of understanding of resources required for case management, psychosocial support, and other skills required. Concerns have been raised about pricing mechanisms leading to mergers and smaller providers being driven out of service delivery. Mendoza warns that “there is a real danger that the focus will be on pricing of services, on eligibility, and on entitlement” (21).
- *Capacity building for service providers, service users, and carers*: it has been argued that service providers will need to take on more market-based approaches and will require capacity building to meet the demands of consumers and the NDIS. Similarly, “mental health consumers” and carers will require capacity building and support to develop packages of care.
- *Assessment*: Concerns have been raised that assessment: would not involve carers, service providers, and other support people; that it would not be transparent; and that assessment tools were not specific for people with a psychiatric disability. NDIA staff were not necessarily specialists and aware of mental health issues and psychosocial disability support needs.
- *Impact on other sectors*: There may be unintended consequences for housing and employment sectors as a result of people with psychiatric disability not receiving support through the NDIS.
- There is a need for psychosocial disability support services to *engage more with clinical mental health services and the legal system*. There were concerns about the implementation of advance directives for care.

There are many opportunities to further explore the implementation of the CRPD with disability for all disability groups, and associated policies and schemes such as the NDIS, but especially for people with psychiatric disability, who may have traditionally missed out on psychosocial disability services or been lower down the “hierarchy of disability” (where some forms of disability are seen as being more acceptable than others) for generic disability services, due to stigma.

The abovementioned concerns about the introduction of the NDIS in Australia highlight the importance of future research, which explores policy and interventions promoting positive rights and social inclusion for people with psychiatric disability, including the broad range of human rights enshrined in the CRPD. This would entail a cross-sectoral approach and engagement across health and social care sectors, as well as with service users and carers. Such research is warranted if we are to move beyond medical and charity models of disability in practice and research, and ensure full social inclusion for people with a psychiatric disability.

## Author Contributions

The author confirms being the sole contributor of this work and approved it for publication.

## Conflict of Interest Statement

The author declares that the research was conducted in the absence of any commercial or financial relationships that could be construed as a potential conflict of interest.

## Footnotes

^1^http://www.adelaidenow.com.au/news/south-australia/ed-patients-waiting-more-than-24-for-a-bed-national-hospital-snapshot-survey-reveals/news-story/23046e940ef1c80df3b841f9bc8e1317.

^2^http://indaily.com.au/news/2016/02/12/mental-health-emergency-room-waiting-times-halved/.

^3^National Disability Insurance Agency. *What is the National Disability Insurance Scheme?* Available from: http://www.australia.gov.au/directories/australia/ndia.

^4^Australian Government. *Conceptual Landscape – the Context for Reform*. Australian Law Reform Commission. Available from: https://www.alrc.gov.au/publications/supported-and-substitute-decision-making.

^5^NDIS. *Guide to service agreements*. Available from: https://www.ndis.gov.au/html/sites/default/files/documents/Service_Agreement.docx.
